# Associations of sexual dysfunction with problematic pornography use and attachment styles: a cross-sectional study of Hungarian-Spanish samples

**DOI:** 10.1192/j.eurpsy.2024.153

**Published:** 2024-08-27

**Authors:** S. Kató, Z. G. Pintér-Eszenyei, M. A. Rando Hurtado, A. K. Csinády, A. Szemán-Nagy

**Affiliations:** ^1^Faculty of Humanities, Institute of Psychology, University of Debrecen, Debrecen, Hungary; ^2^Faculty of Psychology and Speech Therapy, University of Málaga, Málaga, Spain

## Abstract

**Introduction:**

In the last decades, growing evidence suggests, that young adults and even adolescents consume more and more pornographic content, which might lead to behavioural addictions. Excessive pornography use was found to be associated with higher rates of sexual dysfunctions, such as genital dysfunction or disorders related to desire, arousal, orgasm and pain. The role of attachment style on sexual function has still rarely been investigated.

**Objectives:**

To examine associations between sexual dysfunction, problematic pornography use and attachment styles in a Spanish-Hungarian sample.

**Methods:**

A cross-sectional comparative study was carried out in 2023 which included a Hungarian (N=447; 63% female; age: 30,5±9,8) and a Spanish sample (N=201; 72% female; age: 40,7±14) from the general population. In the online survey, we used the Arizona Sexual Experiences Scale (ASEX) to measure sexual dysfunction, the Problematic Pornography Use Scale (PPCS) to assess pornographic content consumption within the theoretical framework of addiction and the Relationships Questionnaire to explore the attachment styles of the subjects.

**Results:**

13% of the Hungarian sample and 19% of the Spanish sample reported severe sexual dysfunction (ASEXTotal >19). The Hungarian sample reported more problems related to orgasm (climax and satisfaction). Overall, 7% of the Hungarian sample and 1% of the Spanish sample reported very severe problems (PPCSTotal >76) with pornography use. We found significant differences in every subscale and the Hungarian sample reportedly showed more difficulties in every aspect, especially in salience and mood change. Regarding attachment styles, the samples also showed significant differences (Hungarian: 31% secure, 26% anxious-ambivalent, 20% avoidant, 23% disorganized; Spanish: 53% secure, 11% anxious-ambivalent, 23% avoidant, 13% disorganized). In the combined sample, secure attachment style was associated with the least difficulties in sexual functioning, whereas subjects with anxious-ambivalent style reported more problems in sexual drive, arousal and erection. Disorganized attachment style was associated with the most severe dysfunction in orgasm (climax and satisfaction). The association between problematic pornography use and attachment styles was more consistent. Secure attachment style showed the least of problems, whereas subjects with anxious-ambivalent and disorganized attachment styles reported the most, especially in salience and mood change.

**Conclusions:**

Our findings showed significant intercultural differences between the two samples and highlighted the potential role of attachment styles in sexual functioning and problematic pornography use. A more profound understanding of the relationship between attachment and sexual functioning could facilitate potential treatment of sexual dysfunctions by addressing attachment issues in psychotherapy.

**Disclosure of Interest:**

None Declared

